# How COVID-19 Broke the Barriers Related to the Implementation of Telecare—Patients’ Experiences with a New form of Providing Health Services in Primary Health Care

**DOI:** 10.3390/healthcare11040497

**Published:** 2023-02-08

**Authors:** Weronika Ciećko, Kinga Labunets, Małgorzata Wojnarowska, Dominika Bosek, Justyna Skwierawska, Tomasz Bandurski, Ewa Bandurska

**Affiliations:** 1 Center for Competence Development, Integrated Care and e-Health, Medical University of Gdańsk, 80-210 Gdańsk, Poland; 2 Division of Internal and Pediatric Nursing, Medical University of Gdańsk, 80-210 Gdańsk, Poland; 3 Students’ Scientific Circle of Economics and Management in Healthcare, Medical University of Gdańsk, 80-210 Gdańsk, Poland; 4 Division of Radiology Informatics and Statistics, Medical University of Gdańsk, 80-210 Gdańsk, Poland

**Keywords:** teleconsultation, primary health care, COVID-19

## Abstract

Background: The COVID-19 pandemic has significantly contributed to accelerating the development of telemedicine and eHealth. The main aim of this study was to assess the attitudes of patients towards remote care implemented in general practice (GP) during the pandemic. Methods: The study was a pilot study conducted in March–April 2021, considering technical aspects of using teleconsultations, and evaluating the difficulties, advantages and disadvantages for patients. When assessing opinions, a simple Likert scale was used, where a value of 1 meant the worst possible assessment or strong disagreement and the upper value the best or full agreement of the respondent. Results: The study covered 408 respondents. Their biggest organizational challenge reaching GPs by telephone, regardless of the patients’ home location (*p* = 0.23698). Obtaining e-documents was not problematic but was rated as worse by men (*p* = 0.048295). Respondents rated the overall effectiveness of teleconsultations more highly if they could speak directly with a doctor (*p* = 0.0005). There were no differences in willingness to recommend teleconsultations based on gender (*p* = 0.2432), place of residence (*p* = 0.7878), age (*p* = 0.290355) or education (*p* = 0.9109), but people assessing the overall effectiveness of telemedicine more highly were more willing to recommend it (*p* = 0.000). Conclusions: Respondents assess teleconsultations in a differentiated way, noticing both positive and negative features of the remote form of providing health services.

## 1. Introduction

As a result of the development of information and communication technologies, access to medical information has increased over the past few decades [1]. At that time, terms related to eHealth began to appear in the literature. Originally, eHealth was defined as “the use of ICT, especially the Internet, to improve and enable the use of health care” [2]. Currently, this term is interpreted more broadly, and its scope has been extended to incorporate health services, including not only providers of these services and recipients, but also entire healthcare systems [3]. The terms eHealth and telemedicine are often used interchangeably. Nevertheless, there is a semantic difference between them, mainly regarding the possibility of the wider use of eHealth, which is not limited to the provision of remote health services, as is the case with telemedicine [4]. Telemedicine is defined as the use of various types of information and communication digital technologies for the exchange of medical information for medical education and the provision of healthcare services remotely. It is used for a number of different activities, ranging from prevention and diagnosis to treatment, monitoring of treatment and rehabilitation. One of the tools offered by telemedicine is teleconsultation, which at the beginning of the pandemic became an alternative to face-to-face consultations in healthcare facilities [5,6,7,8]. The importance and use of telemedicine solutions has been growing for several years, due to the fact that telemedicine increases the availability of specialist health services. This is particularly important for people living in rural areas [4]. In many countries, healthcare systems promote the use of the “digital-first” model, under which consultations on primary health care are provided via telephone or using tele-visual tools, as well as through online “e-consultation” systems. In-person visits are offered to patients in health situations that require direct doctor–patient contact [5]. An undeniable fact is that the COVID-19 pandemic has significantly contributed to accelerating the development of telemedicine and eHealth, both in the world and in Poland [6,7], which has increased public awareness of the existence of such solutions and driven their widespread use in everyday medical practice. Studies on the perception of teleconsultation during the COVID-19 pandemic were conducted in area of different medical specialties, e.g., gynaecology [9] and otolaryngology [10]. The pandemic also accelerated work on the part of the decision makers, which limited the wider possibilities of using teleconsultations before 2020, i.e., especially legal issues and reimbursement for medical procedures [8]. The introduction of teleconsultations greatly facilitated cooperation between the patient and the doctor during the lockdown, and limited in-person access to healthcare facilities. In order to prevent the spread of the SARS-COV-2 virus, in March 2020 patients were allowed to consult their GP (primary health care) doctor in the form of teleconferences, carried out under contract for the provision of healthcare services using ICT systems or other communication systems [11]. During the study period (March–April 2021), teleconsultations were commonly used as a replacement for traditional services where possible. In 2021, 48.6 million teleconsultations were provided in by GPs alone, which accounted for about 40% of all advice provided by GPs [12]. While the use of teleconsultations decreased in the second half of 2021 (mainly due to restrictions introduced by funding body on the age of patients and the type of health problem that could be assessed by a doctor during teleconsultations), they remain an important method of providing services that meet a significant part of the demand for GP services. Currently, teleconsultation is still one of the most easily available and frequently used forms of advisory implementation. In clinical practice, there are guidelines for the design and implementation of health services in the form of teleconsultations, including, among others, handling of patient identification, privacy and consent [13]. One such set of guidelines was developed by the WHO [14] with the aim of supporting Member States throughout the decision-making process on teleconsultations. In connection with the COVID-19 pandemic, and thus the increased use of teleconsultations in clinical practice, their effectiveness and efficiency, and the satisfaction of recipients related to the use of this tool should be the subject of further research.

The main research question in this study was to assess the attitudes towards remote care implemented in general practice during COVID-19 pandemic.

In order to obtain an answer to this question, a study was carried out to assess patient satisfaction related to the use of teleconsultations as part of primary health care. In addition, specific objectives were set, including:

1. Analysis of general parameters related to the use of GPs by Poles during the pandemic;
2. Determining the attitudes of Poles towards teleconsultations and readiness to use them in the future;
3. The identification of strengths and weaknesses in the use of ICT systems or other communication systems in the provision of GP services.

The study of patients’ attitudes towards new tools is a valuable element of building an effective system of providing health services. This study allows an assessment of the level of satisfaction and adaptation of patients to the change, which was the introduction of teleconsultations at the time of the outbreak of the COVID-19 pandemic. The gathering of this data may affect the introduction and modification of previously adopted assumptions regarding the provision of remote health services. In addition, measuring patient satisfaction can help identify gaps in this form of service provision and enable changes and improvements to be made based on them. 

## 2. Materials and Methods

The study was a pilot study and was conducted in March–April 2021, i.e., during one of the COVID-19 waves in Poland. The survey used a proprietary questionnaire consisting of 22 closed questions and a demographics question, which was sent to respondents via Google Forms. The questions concerned five areas:

4. Technical and organizational aspects of the implementation of teleconsultations
5. Evaluation of difficulties in the implementation of selected elements of teleconsultations
6. Overall effectiveness of teleconsultations
7. Opinions on teleconsultations
8. Willingness to recommend teleconsultations

The study also analyzed the most frequently indicated advantages and disadvantages of teleconsultations. 

In the opinion questions assessing the difficulties, advantages and disadvantages, a simple Likert scale was used, where a value of 1 meant the worst possible assessment or strong disagreement of the respondent to a specific statement, and the upper value (depending on question 4 or 5) meant full agreement of the respondent with a specific statement.

All calculations were performed using a Microsoft Excel 2019 spreadsheet and StatSoft Inc., Tulsa, OK, USA, analytics package, Statistica version 13.3. In the descriptive statistics of the quantitative data, classical positional measures such as arithmetic mean and median and standard deviation were used as measures of variability. The normality of the distribution of variables and the equality of variance of the tested feature in groups were tested with the Shapiro–Wilk test and the variance equality test, respectively. When comparing two groups of quantitative data, the Mann–Whitney U test was used in connection with the failure to determine the existence of parametric data with normal distribution and homogeneous variance. In all statistical tests, *p* < 0.05 was assumed as the level of statistical significance of differences.

### Study Group

The study involved 408 people. The majority were women (80.15%; N = 327). The average age was 35.5 years (Me = 27 years). The respondents most often declared that they were single (62.50%; N = 255). The majority of respondents had higher education (53.34%; N = 218). Detailed characteristics of the study group are presented in Table 1.

## 3. Results

### 3.1. Technical Aspects of the Implementation of Teleconsultation

Almost all respondents (98.04%) indicated a telephone conversation as the most common form of consultation provided in the last 12 months. Other forms of communication, e.g., video conference or chat, had occurred only sporadically. During most of the teleconsultations, the patient had contact with a doctor (72.30%). A further 19.85% could received such a call on explicit request. 

**Figure 1:**
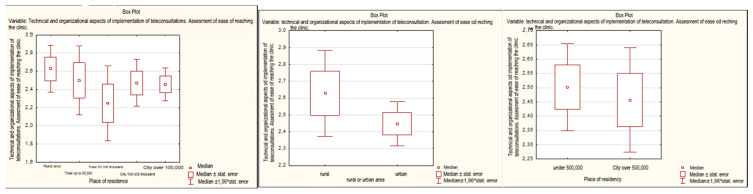
Assessment of ease of getting a call through to a clinic, depending on the place of residence.

It seems that the organizational challenge that deserves the most attention remains the issue of getting through while calling the clinic. This parameter was rated by respondents on a scale from 1 to 5, with 1 being the worst rating and 5 being the best. More than half of the respondents (54.17%) rated this parameter as 1 or 2. These difficulties were similar, regardless of whether the respondents were urban or rural residents (*p* = 0.23698). However, it should be noted that the average results were higher, so the rating was more positive among rural residents than among city residents (2.63 vs. 2.45). The analysis was also made in terms of the number of inhabitants of a given agglomeration. Although the differences were not statistically significant (*p* = 0.707), the assessment was also better in the group of people living in smaller towns (below 500,000 inhabitants)—2.50 vs. 2.46 in the group of residents living in cities >500,000 thousand inhabitants. The biggest problems with getting through when calling the clinic were experienced by respondents living in cities of 50–100,000 inhabitants, and then, with the growing size of the urban agglomeration, these problems seemed to become less acute (Figure 1).

The respondents rated other parameters more positively in terms of assessing the technical aspects of the implementation of teleconsultation, most often giving a score of 4 when assessing the waiting time for teleconsultations (34.07%) and the punctuality of the teleconsultation (29.66%), and most often giving a 5 rating when assessing the quality of the connection (51.47%).

### 3.2. Evaluation of Difficulties in the Implementation of Selected Elements of Teleconsultations

The respondents rated the difficulties on a scale of 1–4, where 4 meant very easy and 1 very difficult. Most of the time, patients did not encounter any significant difficulties during the implementation of teleconsultations, assessing individual aspects most often as very easy (verification of personal data—49.26%; obtaining an e-prescription and/or e-sick leave 50.00%) or easy (comprehensibility of doctor’s recommendations—49.51%; description of own symptoms/ailments—42.65%).

**Figure 2:**
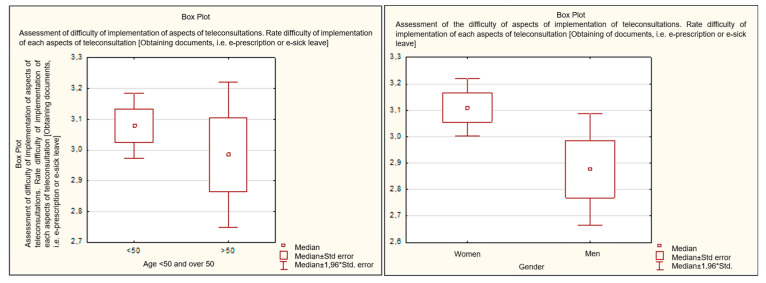
Assessment of the difficulty of obtaining an e-prescription by age and gender.

Obtaining e-documents is a key element of the eHealth system, allowing for obtaining assistance in a completely remote form. This was assessed similarly by respondents regardless of age (analyzed in two groups—above and below 50 years of age, *p* = 0.485). Older people rated this parameter only slightly worse, on average at 2.98 points, compared to people from the younger age group assessing it at 3.08 points. It is worth noting, however, that people from the older age group rated this parameter more diversely, which indicates that some people in this group were experiencing extreme difficulties. Attention is drawn to the fact that women rated the ease of this activity significantly better than men (*p* = 0.048295). In the assessment of women on the 4-point ease scale, this activity was rated at an average of 3.11 points, while the assessment of men was 2.88 points. It is also important to note that men assessed this activity in a more diverse manner (Figure 2).

### 3.3. Overall Effectiveness of Teleconsultation

Using a scale of 1–5, where 1 was the worst and 5 was the best, the respondents first assessed the scale of their difficulty with understanding the information provided by the doctor. The results indicated that this was not a major challenge. In total, less than 13% chose the worst or second worst option (1 or 2). 

The respondents most often determined that the effectiveness of teleconsultations in solving a health problem was the reason for rating the visit positively, assigning a rating of 4 (30.15%), which was the second-best option, or giving the highest possible rating of 5 (25.74%). In total, more than half of the respondents gave the two highest ratings, indicating that teleconsultation had effectively solved their health problem. At the same time, it should be noted that 10.05% of respondents gave the worst rating. As this factor can be considered crucial, a more thorough assessment has been carried out. There were no significant differences in the assessment due to gender (*p* = 0.21) or age analyzed in the two groups, i.e., people over and under 50 years of age assessed this aspect similarly (*p* = 0.7717). 

The sense of safety and comfort was assessed in a more varied way. While the most frequently chosen assessments were 4 and 5 (in total 53.93% of respondents), it is worth mentioning that close to a quarter of the respondents rated this aspect as 1 or 2. However, no differences were found based on gender (*p* = 0.495) or age (*p* = 0.345). It is worth noting, however, that in the group of women, a total of 25% of the respondents gave the lowest scores (1 or 2), while in the group of men this rating was given by 19%. This may indicate different perceptions of this aspect and different needs in this regard. 

**Figure 3:**
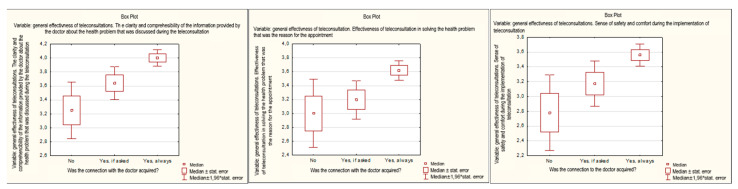
Ability to have a direct conversation with a doctor and the components of the overall assessment of the effectiveness of teleconsultations.

The overall assessment of the effectiveness of teleconsultations (meaning satisfaction with the completed teleconsultation in the opinion of respondents) was carried out for the average number of points obtained in the three aspects discussed above. It was shown that overall assessment was correlated with acquiring connection with a doctor during the teleconsultation. It was shown that the highest rating of total effectiveness was found in the group of people who always had the opportunity to talk to a doctor (*p* = 0.0005). The ability to connect with a doctor was significantly correlated with each of the factors analyzed in the overall effectiveness assessment (Figure 3).

### 3.4. Opinions on Teleconsultation

Opinions were expressed by respondents on a Likert scale ranging from 1–5, where 1 meant completely disagree and 5 meant completely agree. 

The assessment of the effectiveness of teleconsultations compared to traditional appointments was similar, regardless of gender (*p* = 0.34) and age (*p* = 0.48), but its nature was most often negative (in total 63.5% of respondents assigned answers 1 or 2, disagreeing with the thesis about the similar effectiveness of these two forms of performance). In total, 36.76% of the respondents completely disagreed with the statement that they would prefer to have all appointments remotely. However, it should be noted that 18.62% of the respondents were inclined to accept such a possibility (rates 4 and 5), which indicates a different approach to this topic. This may indicate that patients seem to see both restrictions on the exclusive use of teleconsultations in their health consultations (see more in Table 2 in the section about their disadvantages) and the benefits of this option (see more in Table 2 in the section about their advantages).

### 3.5. Willingness to Recommend Teleconsultations

A diversity of opinions can also be seen in the question about the willingness to recommend teleconsultations to one’s friends and family as a convenient, safe and effective form of GP services. This factor was evaluated by the respondents using a Likert scale, where 1 meant completely disagree and 5 meant completely agree. In all, 26.96% of the respondents completely disagreed with this claim and a further 17.89% gave a rating of 2, which also led to the rejection of this claim. However, it should be noted that a total of 34.56% of respondents would be willing to recommend teleconsultations. 

There were no differences in the willingness to recommend teleconsultations based on gender (*p* = 0.2432), or place of residence when analyzed together (*p* = 0.8088), divided into inhabitants of rural and urban areas (*p* = 0.7878), residents of towns with more and fewer inhabitants (*p* = 0.259557), age (*p*= 0.290355), education (*p* = 0.9109) or frequency of use of GP (*p* = 0.401578). It is worth noting, however, that people who used GP services more than six times a year were less likely to recommend teleconsultations than people who used them less frequently (on average 2.64 vs. 2.80 points). 

However, it was shown that people assessing the overall effectiveness of this form of performance more favorably were significantly more willing to recommend it to family and friends (*p* = 0.000), and that all components had a significant impact on willingness to recommend. The most strongly correlated factor (r = 0.607) was the sense of safety and comfort during the implementation of the teleconsultation. The details are shown in Table 3.

### 3.6. Advantages and Disadvantages of Teleconsultations

The respondents also assessed the advantages and disadvantages of teleconsultations (Table 2). Worth noting is the opinion of the respondents indicating that teleconsultation does not, in their opinion, provide them with greater mental comfort than traditional appointment. This factor was analyzed in detail in the previous part of the paper. 

The respondents were divided in terms of opinions on this subject and although there were no significant differences due to gender, it was noted that women were more likely to assess this parameter negatively.

This section may be divided by subheadings. It should provide a concise and precise description of the experimental results, their interpretation, as well as the experimental conclusions that can be drawn.

## 4. Discussion

Before the COVID-19 pandemic, the use of telemedicine solutions was not as common as during its duration [15]. In Poland, the perception of teleconsultations in various contexts was analyzed. These studies concerned, for example, the perspective of service providers [16,17], but also of patients [18]. The study by Kludacz-Alessandri et al. [19] assessed how the use of teleconsultations affected the quality of communication between the GP and the patient. In the study by Stepaniuk et al. [20], technical and organizational factors related to teleconsultations were assessed. In this study, the respondents had doubts about guaranteeing a sense of security and comfort during teleconsultation, as well as similarly related to the limitations of teleconsultation in terms of conducting a physical examination. Interestingly, while in the presented study the respondents highly rated both the quality of the connection and the clarity and comprehensibility of information shared by the doctor, in another Polish study [21] only 7.5% of students rated the quality of doctor–patient communication as high. In Poland, negotiations with the public payer regarding teleconsultations took place in 2019, but it was the outbreak of the pandemic that made them possible from March 2020 [11]. A similar trend can be noted in other countries: in the United States in 2016, only 15% of doctors worked in health facilities that used tools enabling the provision of remote services [22,23]. Making medical appointments via digital tools aroused anxiety and a sense of lack of credibility, both among patients and healthcare providers. The rapid spread of the virus and the increase in the number of cases and deaths contributed to the introduction of telemedicine in real-life medical practice. This was the only alternative to personal visits, which, due to the pandemic situation, were not possible to the same extent as before. Teleconsultations have started to play an important role in increasing the availability of primary health care, securing a significant part of the demand for these services. The sudden development of eHealth and the use of telemedicine tools have highlighted the disadvantages of this solution. Tools that had been developed for many years and were not put into use before the outbreak of the pandemic were suddenly implemented into the healthcare system and it was necessary to quickly learn how to use them in reality. Among the main barriers to telemedicine, technical aspects are most often reported. A systematic review of Ftouni 2022 indicated that 21 studies had addressed this issue [24]. This is confirmed by the results of this survey, in which 35% of respondents declared that the biggest disadvantages of teleconsultations were hardware limitations and difficulty in getting through to the clinic. This limitation was declared both by the respondents to this survey, as well as by the respondents to the survey conducted by the National Health Fund and the Ministry of Health of Poland [25]. In addition, the respondents declared their lack of future willingness to use services provided by the GP only in the form of teleconsultations. An inherent element of the limitations and barriers to the use of telemedicine solutions, including teleconsultations, is the issue of digital exclusion, which concerns the division of society into people who have and are able to use the Internet and various forms of digital communication, and those with insufficient skills. Digital exclusion depends on age and gender. As indicated in the 2017 Narasimha study, telemedicine system designers should take into account differences in the ability of elderly people to use such systems [26]. In addition, the Eberly 2020 study indicated that less affluent people, the elderly, and women were less likely to use teleconsultations [27]. Despite the limitations of the use of telemedicine solutions in the group of elderly patients, this barrier is not an impassable one. In the study conducted by Buliński et al., the openness of this social group to the offered telemedicine solutions was analyzed. The results of the study indicated that the challenge seems to be the attitudes of older people towards, for example, teleconsultations, i.e., their habits, rather than the digital exclusion itself. Despite the visible limitations, in this study approx. 40% of respondents were in favor of this form of providing services and declared that they would recommend teleconsultations as a convenient, effective and safe form of providing GP services. This coincides with the findings of other authors, who point out the fact that despite concerns and reservations, some patients perceive teleconsultations positively and are willing to continue to use them in the future as one of the forms of visits [19,28]. The results obtained in this study coincide with other studies in which it was proven that some patients value personal contact with a doctor, primarily due to the possibility of conducting a full physical examination and due to higher physical and mental comfort than in the case of remote visits [19,29,30,31]. One of the aspects that indirectly relate to the subject of discussion is the issue of feeling safe and mentally comfortable during teleconsultations. The respondents in this study assessed it differently, at the same time it is noteworthy that women scored lower than men. It is known that women and men use telecare with different frequencies [32] and perceive its role differently [33]. However, the results of research on the level of satisfaction with telecare depending on gender are not unequivocal [34]. For example, in the study by Mason et al., it was shown that, while gender affects the perception of the benefits of patient-centric care, in the case of telemedicine assessment, these differences are already bordering on statistical significance [35]. In turn, in the study by Bagchi et al., women had the lowest levels of comfort associated with telecare use, and the differences identified between the sexes (male, female and transgender) were statistically significant [36]. Invariably, most studies indicate the need for further analysis in identifying and describing risks to patient safety in the area of telecare, in order to better understand how to avoid and minimize potential harm to patients [37] and how to prevent the marginalization of the patient’s role [38].

The described form of providing services was appreciated by the patients of this study, which is consistent with the results of other researchers. However, it seems that in Poland it would be necessary to use other ways of contact with the patient than by telephone, as this is associated with higher levels of satisfaction [28]. They see the advantages of teleconsultations, which mainly include epidemiological safety issues and time saving. Most patients in this study positively assessed the technical implementation of the elements of telemedicine, despite the fact that this form of service provision was not previously used in the Polish healthcare system. Nevertheless, telemedicine cannot replace personal medical care for all patients—it is undoubtedly not to be used in the case of severe diseases, for which a thorough examination requires a personal visit and a thorough physical examination. Although this topic has been discussed by researchers for many years, the biggest challenge deserving special attention is still the difficulty in getting through to the clinic, which coincides with the findings of other researchers [25,39]. The supply side seems to be well prepared to provide remote services. The course of the teleconsultation was positively assessed in technical terms. The respondents mostly defined its individual stages (such as verifying personal data, obtaining information about the disease by the patient, comprehensibility of medical recommendations) as easy or very easy.

## 5. Conclusions

Telemedicine tools, including teleconsultations, can play a significant role in the healthcare system, enabling patients to access healthcare services and, in the event of epidemic/pandemic exposure, protecting both them and doctors from exposure to viruses. Respondents to this survey are ready to recommend teleconsultations as a convenient and safe form of providing health services, therefore, the potential for further use of remote consultation can be noticed. Respondents believe that teleconsultation is a form of care that saves time, so it can be concluded that from the perspective of respondents they are time effective. In addition, in the face of epidemic exposure, this form of medical visits is convenient and safe. The most important limitations of teleconsultations include the difficulty in getting through to the clinic. The greatest attention should be paid to the issue related to the lack of physical examination. This limitation is the biggest problem for the respondents to this survey. Teleconsultations introduced as a result of the COVID-19 pandemic should be continued under standard non-pandemic conditions. However, the level of satisfaction and doubts of patients in relation to the level of services provided should be taken into account.

## 6. Limitations

The limitation of this study is the study group, which includes a small number of people with similar characteristics. The small size of the surveyed sample results from technical and organizational issues. The study was conducted during the pandemic, which made it difficult for the team to obtain more responses. However, this is a pilot study that would be worth extending on a larger scale for a broader analysis. Further research on issues related to the accessibility and use of teleconsultations by the public is needed.

## Figures and Tables

**Table 1:** Characteristics of the study group.

Feature	N = 408
Age (years)	
Average	35.52
Standard deviation	14.61
Median	27
Minimum/Maximum	17/87
Age (N; %)	
Under 50 years of age	342; 83.82%
Over 50 years of age	66; 16.18%
Gender, (N;%)	
Women	327; 80.15%
Men	81; 19.85%
Place of residence (N;%)	
Rural area	78; 19.12%
Town up to 50,000 residents	42; 10.29%
City of 50–100 thousand inhabitants	28; 6.86%
City of 100–500 thousand inhabitants	87; 21.32%
City over 100 thousand inhabitants	173; 42.40%
Education (N;%)	
Primary or junior high school	4; 0.98%
Professional	15; 3.68%
High school	171; 41.91%
Higher education	218; 53.43%
Marital Status (N;%)	
Married	138; 33.82%
Widow/Widower	4; 0.98%
Single	255; 62.50%
Divorced/Separated	11; 2.70%
Having offspring (N;%)	
Yes	137; 33.58%
No	271; 66.42%
Frequency of GP visits (N;%)	
1–2× per year	172; 42.16%
3–6× per year	170; 41.67%
6–12× per year	59; 14.46%
more than 12 times a year (i.e., more than once a month)	7; 1.72%

**Table 2:** Potential advantages and disadvantages of teleconsultations in the opinion of the respondents (number and %).

Advantages and Disadvantages of Teleconsultations	The Most Frequently Indicated Response	Number	%
Advantages:			
Teleconsultations provide easier access to doctors, a greater choice of specialists	Rather yes	140	34.31%
Teleconsultations provide short waiting times for appointment	Rather yes	164	40.39%
Teleconsultations provide greater mental comfort than when talking in the office	Rather not	182	44.61%
Teleconsultation is convenient, no need to leave home, no travel costs	Definitely yes	199	48.77%
Teleconsultations increase epidemiological safety	Definitely yes	220	53.92%
Teleconsultation is the absence of the need to reschedule at work or to leave work in order to go to the clinic	Definitely yes	192	47.06%
Disadvantages:			
Teleconsultation is a hardware limitation, difficulty in accessing the clinic	Definitely yes	144	35.29%
Lack of physical examination and related fears that the doctor will make the wrong diagnosis	Definitely yes	297	72.79%
Unintelligible doctor’s recommendations, communication problems with the doctor	Rather not	206	50.49%
Lack of information from the clinic at what time the doctor will call or appointment at different time than originally designated	Rather not	124	30.39%

**Table 3:** Table of correlations of the components of the overall assessment of effectiveness and willingness to recommend teleconsultation to family and friends.

	N	Mean	Std. Dev.	r (X, Y)	R^2^	*p*
I am ready to recommend teleconsultation to my friends and/or family as convenient	407	2.77	1.41			
Clarity and comprehensibility of information shared by the doctor about related health issue		3.87	1.07	0.49	0.24	0.00
Effectiveness of teleconsultation in solving the health problem which was the reason for the appointment		3.49	1.28	0.55	0.30	0.00
Sense of safety and comfort during teleconsultation		3.42	1.35	0.61	0.37	0.00

## Data Availability

Not applicable.
